# Role of the Insula and Vestibular System in Patients with Chronic Subjective Dizziness: An fMRI Study Using Sound-Evoked Vestibular Stimulation

**DOI:** 10.3389/fnbeh.2015.00334

**Published:** 2015-12-09

**Authors:** Iole Indovina, Roberta Riccelli, Giuseppe Chiarella, Claudio Petrolo, Antonio Augimeri, Laura Giofrè, Francesco Lacquaniti, Jeffrey P. Staab, Luca Passamonti

**Affiliations:** ^1^Centre of Space BioMedicine, University of Rome Tor VergataRome, Italy; ^2^Laboratory of Neuromotor Physiology, IRCCS Santa Lucia FoundationRome, Italy; ^3^Department of Medical and Surgical Sciences, University “Magna Graecia,”Catanzaro, Italy; ^4^Department of Experimental and Clinical Medicine, University “Magna Graecia,”Catanzaro, Italy; ^5^Department of Systems Medicine, University of Rome Tor VergataRome, Italy; ^6^Department of Psychiatry and Psychology, Mayo ClinicRochester, MN, USA; ^7^Institute of Bioimaging and Molecular Physiology, National Research CouncilCatanzaro, Italy; ^8^Department of Clinical Neurosciences, University of CambridgeCambridge, UK

**Keywords:** sound-evoked vestibular stimulation, STBs, fMRI, chronic subjective dizziness, CSD, insula, hippocampus

## Abstract

Chronic subjective dizziness (CSD) is a common vestibular disorder characterized by persistent non-vertiginous dizziness, unsteadiness, and heightened sensitivity to motion stimuli that may last for months to years after events that cause acute vestibular symptoms or disrupt balance. CSD is not associated with abnormalities of basic vestibular or oculomotor reflexes. Rather, it is thought to arise from persistent use of high-threat postural control strategies and greater reliance on visual cues for spatial orientation (i.e., visual dependence), long after triggering events resolve. Anxiety-related personality traits confer vulnerability to CSD. Anomalous interactions between the central vestibular system and neural structures related to anxiety may sustain it. Vestibular- and anxiety-related processes overlap in the brain, particularly in the insula and hippocampus. Alterations in activity and connectivity in these brain regions in response to vestibular stimuli may be the neural basis of CSD. We examined this hypothesis by comparing brain activity from 18 patients with CSD and 18 healthy controls measured by functional magnetic resonance imaging during loud short tone bursts, which are auditory stimuli that evoke robust vestibular responses. Relative to controls, patients with CSD showed reduced activations to sound-evoked vestibular stimulation in the parieto-insular vestibular cortex (PIVC) including the posterior insula, and in the anterior insula, inferior frontal gyrus, hippocampus, and anterior cingulate cortex. Patients with CSD also showed altered connectivity between the anterior insula and PIVC, anterior insula and middle occipital cortex, hippocampus and PIVC, and anterior cingulate cortex and PIVC. We conclude that reduced activation in PIVC, hippocampus, anterior insula, inferior frontal gyrus, and anterior cingulate cortex, as well as connectivity changes among these regions, may be linked to long-term vestibular symptoms in patients with CSD. Furthermore, altered connectivity between the anterior insula and middle occipital cortex may underlie the greater reliance on visual cues for spatial orientation in CSD patients relative to controls.

## Introduction

Chronic subjective dizziness (CSD) or persistent postural-perceptual dizziness (PPPD), as it is defined in the beta draft version of the International Classification of Diseases, 11th edition (ICD-11, http://www.who.int/classifications/icd/en/), is a complex disorder encountered clinically at the interface of neurology, otology, and psychiatry. It is characterized by a sensation of non-vertiginous dizziness and unsteadiness that is typically present throughout the day on most days for at least 3 months (Staab, 2012). CSD symptoms are usually exacerbated by upright posture, patient's own movements, exposure to busy visual environments (e.g., shopping malls), or by the execution of precision visual tasks (e.g., reading from printed pages or electronic screens). CSD may be precipitated by neuro-otological, psychiatric, and other medical conditions that cause acute attacks of vertigo, unsteadiness, or dizziness (Staab and Ruckenstein, 2007). However, these events trigger rather than cause CSD and are not sufficient to sustain the disorder.

Recent research has also begun to reveal the psychological factors that predispose and perpetuate CSD (Staab and Ruckenstein, 2003, 2005). The anxiety-related personality traits of neuroticism and introversion were significantly associated with CSD but not with other disorders that caused similar levels of chronic vestibular symptoms (Staab et al., 2014). Prospective studies found that acute anxiety and hypervigilance about vestibular symptoms were strong predictors of persistent dizziness after acute vestibular events (Godemann et al., 2005; Heinrichs et al., 2007). Persistent dizziness was also associated with visual dependence (Cousins et al., 2014). Furthermore, a recent functional magnetic resonance imaging (fMRI) study in healthy volunteers exposed to sound-evoked vestibular stimulation showed that high neuroticism and introversion scores were associated with changes in activity and connectivity in brain areas belonging to vestibular and anxiety circuits (Indovina et al., 2014). These data support anatomical and clinical models which suggested that anxiety and anxiety-related personality traits modulate responses within vestibular and emotional systems in the brain (Balaban and Thayer, 2001; Staab et al., 2013).

The core region of the multimodal vestibular cortex has previously been defined as the parieto-insular vestibular cortex (PIVC) (Guldin and Grüsser, 1998; Brandt and Dieterich, 1999; Indovina et al., 2005; Lopez and Blanke, 2011; Lopez et al., 2012; zu Eulenburg et al., 2012; Lacquaniti et al., 2013). Imaging studies showed that the human PIVC includes part of the posterior insula and parietal operculum, as well as part of the temporo-peri-sylvian cortex in the superior temporal gyrus (Bense et al., 2001; Bottini et al., 2001; Lopez and Blanke, 2011; zu Eulenburg et al., 2012; Lacquaniti et al., 2013). Reports of intra-surgical electrical stimulation or epileptic discharges in these peri-sylvian areas described strong sensations of dizziness, rocking or swaying, which were qualitatively similar to cardinal symptoms of CSD (Penfield, 1957; Blanke et al., 2002; Isnard et al., 2004; Nguyen et al., 2009; Mazzola et al., 2014). Additional functional investigations showed that vestibular signals reached the anterior insula, which has a regulatory role on interoceptive inputs (Craig, 2009; Gu et al., 2013), as well as the adjacent inferior frontal gyrus and anterior cingulate cortex (Shinder and Taube, 2010; Hüfner et al., 2011; Lopez and Blanke, 2011; Lopez et al., 2012; Hitier et al., 2014; Dieterich and Brandt, 2015). Strong reciprocal connections were identified between posterior and anterior insula and perisylvian regions, frontal opercula, hippocampus and anterior cingulate cortex (Deen et al., 2011; Almashaikhi et al., 2014a,b). The hippocampus may also be particularly important for vestibular-anxiety system interactions, given its ability to process vestibular inputs for spatial orientation (Dieterich and Brandt, 2015) and its role in adding context to noxious experiences to extinguish aversive conditioning (LeDoux, 2000; Grillon, 2002; Kalisch et al., 2006; Ji and Maren, 2007; Alvarez et al., 2008; Indovina et al., 2011; Baldi and Bucherelli, 2015). The latter function may be particularly relevant in CSD. Recent pathophysiologic models suggested indeed that CSD patients fail to return to normal relaxed postural control strategies following their use of high-threat postural responses (e.g., stiffening of stance) that are transiently activated during balance challenges (Staab, 2012, 2014; Staab et al., 2013).

Visual cortical areas are also critical for spatial orientation and could consequently play a key pathogenic role in CSD. Imaging studies showed reduced activation of visual cortices during vestibular stimulation (Brandt et al., 1998; Brandt, 1999; Bense et al., 2001), and this was hypothesized to be a cortical mechanism contributing to stabilize visual images during self-motion, along with brainstem reflexes (such as the vestibulo-ocular reflex). In line with this view, we have recently found that the functional interplay between visual and vestibular regions was altered in favor of higher visual cortical activity in people with high levels of neuroticism when exposed to sound-evoked vestibular stimuli (Indovina et al., 2014). This shift may represent the cortical substrate of visual dependence, that consists of greater reliance on visual cues for spatial orientation and was associated with the type of persistent dizziness seen in CSD (Cousins et al., 2014).

The present study was aimed at elucidating whether the brain regions that process vestibular, visual, and anxiety-related information show anomalous activity or altered connectivity patterns in response to vestibular stimulation in CSD patients relative to controls. To accomplish this, we used a previously developed paradigm that employs sound-evoked vestibular stimulation (Janzen et al., 2008; Schlindwein et al., 2008; Indovina et al., 2014, 2015). We hypothesized that, relative to controls, patients with CSD would show altered functioning in brain regions that are thought to be important in CSD. These include vestibular cortical areas, particularly PIVC (Indovina et al., 2005; Lopez and Blanke, 2011; Lopez et al., 2012; zu Eulenburg et al., 2012), visual cortical areas (Cousins et al., 2014; Indovina et al., 2014), and regions of potential interaction between vestibular and anxiety systems, such as the hippocampus, anterior insula, inferior frontal gyrus, and anterior cingulate cortex (LeDoux, 2000; Grillon, 2002; Kalisch et al., 2006; Etkin and Wager, 2007; Alvarez et al., 2008; Craig, 2009; Shinder and Taube, 2010; Hüfner et al., 2011; Indovina et al., 2011; Lopez and Blanke, 2011; Lopez et al., 2012; Gu et al., 2013; Staab et al., 2013; Hitier et al., 2014; Dieterich and Brandt, 2015).

To focus on core brain mechanisms of CSD and minimize confounds from psychological risk factors (i.e., anxiety-related personality traits) and comorbidity (i.e., anxiety and depressive disorders), we matched CSD patients and healthy controls on standardized measures of neuroticism, introversion, anxiety and depression, and repeated the analyses after excluding the participants with active psychiatric disorders.

## Methods

### Participants

Eighteen CSD patients, and 28 healthy volunteers (26 were from the previous study Indovina et al., 2014) gave written informed consent to participate in this study, which was approved by the Santa Lucia Foundation Research Ethics Committee in Rome, according to the Helsinki declaration (http://www.wma.net/en/30publications/10policies/b3/). Eighteen healthy volunteers were included in the control group, while 10 were included in the PIVC localizer group (see below). All participants were right-handed, as assessed via the Edinburgh Handedness Inventory (Oldfield, 1971).

Participants also completed a series of questionnaires. To measure personality traits of the five Factor Model (neuroticism, extraversion, openness, agreeableness, conscientiousness), they completed a computerized version of the Italian translation of the Revised NEO Personality Inventory (NEO-PI-R) (Costa and McCrae, 1997). The Mini-International Neuropsychiatric Interview (MINI) (Sheehan et al., 1998), Generalized Anxiety Disorder questionnaire (GAD7) (Spitzer et al., 2006), and Patient Health Questionnaire for depression (PHQ9) (Kroenke et al., 2001) were used to assess for psychiatric comorbidities. Finally, the severity of dizziness symptoms in CSD patients was measured with the Dizziness Handicap Inventory (DHI) (Jacobson and Newman, 1990).

### CSD patients

Diagnostic criteria for CSD were as follows: (1) persistent non-vertiginous dizziness, unsteadiness, or both, lasting 3 months or more, (2) symptoms present most days, throughout the day (though they may wax and wane), (3) symptoms exacerbated by upright posture, exposure to moving or complex visual stimuli, and active or passive head motion (ICD-11, http://www.who.int/classifications/icd/en/). Exclusion criteria included active neuro-otologic disorders other than CSD, chronic medical illnesses, pregnancy, medication use, smoking, and history of head injury. History of quiescent or fully compensated vestibular peripheral deficits at the time of study was not an exclusion criterion. Otogenic illnesses are known to be the most common triggers of CSD (Staab and Ruckenstein, 2003, 2007), and this was the case in our patient sample as vestibular neuritis (*N* = 12), benign paroxysmal positional vertigo (*N* = 4) or both (*N* = 2) represented the main triggers of CSD. These disturbances were localized on the right side (*N* = 10), left side (*N* = 7), or bilaterally (*N* = 1).

CSD patients with previous vestibular neuritis also underwent caloric test in the acute stage of their peripheral vestibular disease and 6 months later. The percentage of reduced vestibular response in the electronystagmography was calculated using the Jongkees' formula (Furman and Jacob, 1993), which revealed mild to medium unilateral canal paresis (relative vestibular reduction in the nystagmus slow-phase velocity peak) across patients in the acute stage (mean = 35%, range 25–45%) and return to normal values 6 months later (mean = 13%, range 5–20%). Patients had persistent CSD symptoms for 33 ± 34 months (mean ± SD), ranging from 8 to 120 months. DHI score for CSD patients was 41 ± 22 (mean ± SD), ranging from 10 to 84.

### Participants' group composition

In a first analysis, 18 CSD patients were compared to 18 healthy volunteers who were matched for demographic variables, personality traits, and anxiety and depression measures (see Table 1). In a follow-up analysis, we also excluded 7 CSD patients and 1 control participant who showed active psychiatric comorbidities when assessed with the MINI (Tables 2, 3).

**Table 1:** **Demographic and clinical characteristics in CSD patients and healthy controls**.

**Demographic and clinical measures**	**CSD patients (*n* = 18)**	**Healthy controls (*n* = 18)**	**Group differences**
	**Mean ± SD**	**Mean ± SD**	***T***, χ^2^, ***P*****-values**
Sex (males, females)	8M, 10F	7M, 11F	χ^2^ = 0.11, *P* = 0.74
Age (years)	35 ± 13	31 ± 6	*T* = 1.4, *P* = 0.18
GAD7 (state-anxiety)	9 ± 5	7 ± 5	*T* = 1.2, *P* = 0.23
PHQ9 (depression)	8 ± 5	6 ± 5	*T* = 1.5, *P* = 0.14
Neuroticism	59 ± 12	53 ± 11	*T* = 1.4, *P* = 0.17
Extraversion	50 ± 8	53 ± 10	*T* = 0.9, *P* = 0.36
Openness	46 ± 10	53 ± 9	*T* = 2.0, *P* = 0.06
Agreeableness	44 ± 9	47 ± 8	*T* = 1.1, *P* = 0.30
Conscientiousness	47 ± 10	51 ± 9	*T* = 1.1, *P* = 0.28

*CSD, chronic subjective dizziness; GAD7, Generalized Anxiety Disorder questionnaire; PHQ9, Patient Health Questionnaire*.

**Table 2:** **List of the active psychiatric conditions in the group of patients with chronic subjective dizziness (CSD) and healthy controls**.

**Subject identifier**	**Anxiety**	**Panic attacks**	**Agoraphobia**	**PTSD**
CSD #1				x
CSD #8	x	x	x	
CSD #15	x	x	x	
CSD #11		x	x	
CSD #7		x		
CSD #10		x		
CSD #17		x		
CSD #14	x			
HC #5			x	x

*PTSD, Post-Traumatic Stress Disorder*.

**Table 3:** **Demographic and clinical characteristics in patients with CSD and healthy controls after removing the 7 CSD patients and 1 control that showed the presence of the active psychiatric comorbidities listed in Table 2**.

**Demographic and clinical measures**	**CSD patients (*n* = 11)**	**Healthy controls (*n* = 17)**	**Group differences**
	**Mean ± SD**	**Mean ± SD**	***T***, χ^2^, ***P*****-values**
Sex (males, females)	5M, 6F	7M, 10F	χ^2^ = 0.05, *P* = 0.82
Age (years)	35 ± 14	31 ± 6	*T* = 0.8, *P* = 0.41
GAD7 (state-anxiety)	8 ± 5	7 ± 6	*T* = 0.79, *P* = 0.43
PHQ9 (depression)	8 ± 5	5 ± 5	*T* = 1.26, *P* = 0.22
Neuroticism	55 ± 9	53 ± 11	*T* = 0.38, *P* = 0.70
Extraversion	51 ± 8	53 ± 10	*T* = 0.49, *P* = 0.70
Openness	49 ± 8	52 ± 10	*T* = 0.91, *P* = 0.37
Agreeableness	43 ± 10	47 ± 8	*T* = 1.1, *P* = 0.26
Conscientiousness	47 ± 7	51 ± 9	*T* = 1.4, *P* = 0.16

*CSD, chronic subjective dizziness; GAD7, Generalized Anxiety Disorder questionnaire; PHQ9, Patient Health Questionnaire*.

### fMRI task

The stimuli were administered via piezo-electric MRI compatible headphones (NordicNeuroLab, http://www.nordicneurolab.com/Products_and_Solutions/fMRI_Hardware/AudioSystem.aspx). Short Tone Bursts (STB) stimuli had a frequency of 500 Hz, rise and fall times of 1 ms, plateau time of 8 ms, and were presented at repetition rates of 3 Hz, as recommended in previous studies (Cheng and Murofushi, 2001a,b; Akin et al., 2003). Previous studies demonstrated that unilateral and bilateral ear stimulation with STB stimuli at 100 dB root mean square (RMS) sound pressure level (SPL) activates the ipsilateral otolith receptors and generates vestibular evoked myogenic potentials (VEMPs) in sternocleidomastoid muscles ipsi- and contralateral to the stimulated ear (Colebatch et al., 1994; Maes et al., 2009; Rosengren et al., 2010; Indovina et al., 2014, 2015; Papathanasiou et al., 2014; Todd et al., 2014). Critically, the same STB stimuli delivered at 65 dB SPL do not evoke VEMPs (Todd et al., 2014). There is also evidence that STBs at 100 dB, but not at 65 dB, evoke responses in a series of vestibular cortical regions including PIVC and posterior insula (Janzen et al., 2008; Schlindwein et al., 2008; Indovina et al., 2014). Hence, to investigate how CSD might influence reactivity within the vestibular system, we compared responses to identical STBs at 100 dB and 65 dB SPL. To control for non-specific brain responses to sound, we also employed white noise stimuli at 100 dB SPL (Indovina et al., 2014, 2015). White noise stimuli were sinusoidally modulated signals that reproduced the time variability and the SPL of STBs (3 peaks per second, 100 dB RMS SPL). This prevented habituation to white noise stimuli.

It was also unlikely that the vestibular stimulation related to the MRI scanner itself (Roberts et al., 2011) could influence our results, as this form of spurious vestibular stimulation is known to activate the canals rather than otolith receptors. Furthermore, any possible spurious vestibular stimulation derived from the MRI scanner would be removed from the main contrast of interest (i.e., STB 100 dB > STB 65 dB), because this stimulation would be constant across brain image acquisitions.

The experimental task included 4 stimuli: (1) STB 100 dB SPL; (2) STB 65 dB SPL; (3) white noise 100 dB SPL; and (4) rest periods with no stimulus presentation. Forty-five stimuli per each type were grouped in blocks lasting 15 s each (rest blocks also lasted 15 s). Four sessions (2 for the left and 2 for the right ear) including 4 blocks per stimulus type were presented in approximately 16 min. The order of sessions and side of first ear stimulated were counterbalanced across participants. To attenuate interferences from external MRI-related noise, we isolated the headphones with soundproof foam cushions and used foam plugs in the non-stimulated ear, as in previous research (Janzen et al., 2008; Schlindwein et al., 2008; Indovina et al., 2014). Throughout the experiment, participants were asked to fixate a central point at the center of the screen, and were told to attend to auditory stimuli and report how many types of stimuli they heard at the end of the experiment.

### Image acquisition

fMRI was performed on a 3-Tesla scanner with an eight-channel head coil. Head movements were minimized using foam pads around participants' head and neck. Whole-brain fMRI data were acquired using echo planar images (EPI) sensitive to blood oxygenation level-dependent (BOLD) contrast (39 axial slices, 3-mm thickness each; repetition time = 2000 ms; echo time = 30 ms; voxel size: 3 × 3 × 3 mm).

### Image pre-processing

Data were pre-processed using SPM8 (http://www.fil.ion.ucl.ac.uk/spm/). EPIs were realigned to the first scan by rigid body transformations to correct for head movements. Realigned scans were normalized to the standard template in the Montreal Neurological Institute (MNI) space using linear and non-linear transformations, and finally images were smoothed with a Gaussian kernel of full width at half maximum of 8 mm.

### fMRI analysis of regional responses

For each participant, a general linear model (GLM) assessed regionally specific effects of task parameters on BOLD activations. First-level GLMs included four experimental conditions (STB100 dB, STB65 dB, white noise 100 dB, and rest) modeled as epochs of fixed duration and convolved with the hemodynamic response function, and six realignment parameters as effects of no interest to remove residual motion-related variance. Low-frequency signal drift was eliminated using a high-pass filter (cut-off, 128 s). An autoregressive model (AR[1]) was applied to correct for voxels' autocorrelations.

Subjects' specific contrast images (STB100 dB > STB65 dB) were generated separately for the right and left ear stimulation, and entered into second-level GLMs investigating: (1) the main effect of stimuli; (2) the main effect of stimulation side; (3) the main effect of group, and (4) any possible interaction effect between (1), (2), and (3). Because white noise stimuli were qualitatively different from STBs, they were not directly included in the comparison exploring the brain activations associated with vestibular stimulation (i.e., the STB100 dB > STB65 dB contrast). Instead, white noise 100 dB > rest contrasts, separately for left and right ear stimulation, were used to exclusively mask the STB100 dB > STB65 dB comparison. In this way, we eliminated residual auditory activity that was present in the STB100 dB > STB65 dB contrast as a result of differences in stimulus loudness. To create the exclusive mask from the white noise 100 dB > rest contrast, we used a lenient threshold of *P* ≤ 0.05, uncorrected. This threshold was chosen to exclude non-specific auditory voxels from the STB100 dB > STB65 dB comparison using a conservative approach.

To assess the statistical significance of the comparisons between healthy controls and patients with CSD we employed a region of interest (ROI) approach using a threshold of *P* ≤ 0.05, controlling for Family Wise Error (FWE) in small volumes (small volume correction, svc). This standard, commonly employed procedure ensures robust protection against both type I errors (false positive results) and type II errors (false negative results) particularly in ROIs for which there was strong a priori hypothesis (Worsley et al., 1996; Friston, 1997).

To localize the PIVC we analyzed an independent group of 10 healthy participants (5 females, 5 males, mean age 31 ± 6 years) using the same procedures described thus far. This was done to avoid circularity in the analysis (Kriegeskorte et al., 2009). Left and right ROIs for PIVC were obtained at a threshold of *p* < 0.05 corrected for multiple comparisons at cluster level (p-FWE-CL corrected, with minimum cluster size estimated at *p* < 0.001) considering the whole brain as the volume of interest (Worsley et al., 1996). The posterior and anterior insulae were also defined as anatomical ROIs from the automated anatomical labeling atlas (aal) (Tzourio-Mazoyer et al., 2002) including only the region posterior and anterior to the central insular sulcus, respectively.

The remaining ROIs were defined as in a previous study (Indovina et al., 2014). In particular, the hippocampus, inferior frontal gyrus (IFg), anterior cingulate cortex (ACC) and occipital cortex were defined as anatomical ROIs using aal. ROIs in the visual cortex included primary and secondary visual cortices (V1 plus V2). Furthermore, the middle temporal gyrus (as defined in the aal template), included the middle temporal motion area which is a highly specialized region for visual motion processing (Kolster et al., 2010). Because specific anatomical ROIs for cerebellar fastigial nuclei were not available, we used a 8-mm radius sphere centered on coordinates reported in Dimitrova et al. (2002) and Indovina et al. (2014).

### Task-dependent functional connectivity analyses: Psycho-Physiological interaction (PPI) in GLMs

To investigate how CSD modulated brain functional connectivity patterns, we used Psycho-Physiological Interaction (PPI) analyses. A PPI represents the change in connectivity between couples of regions that is induced by a specific task (Friston et al., 1997). We sought to identify brain target regions that had differential connectivity with a series of seed regions as a function of processing vestibular stimuli and the diagnosis of CSD (Passamonti et al., 2008, 2009, 2012). We chose the seed regions that showed a differential response to vestibular stimulation in CSD patients relative to controls after excluding those participants that showed active psychiatric conditions (posterior insula and superior temporal gyrus, anterior insula and inferior frontal gyrus, hippocampus, and anterior cingulate cortex, see Results).

Time-series from all participants' seeds were computed using the eigenvariates from all voxels' time series within a 8-mm sphere, and then deconvolved to estimate neuronal time series (Gitelman et al., 2003). PPI analyses were carried out for the contrast STB100 dB > STB65 dB, using right and left ear stimulation data and each of the previously described seeds. PPI regressors were calculated as element-by-element products of the seed region neuronal time series and a vector coding the main effect of task (i.e., 1 for STB100 dB, −1 for STB65 dB). First-level GLMs included the main effect of task and 6 movement parameters as effects of no interest. Subject-specific PPI contrast images were computed and finally entered into second-level GLMs that identified brain regions for which the connectivity with the seeds (for the contrast STB100 dB > STB65 dB, exclusively masked by the white-noise 100 dB > rest) differed between CSD patients and healthy controls. As for the regional analyses, the significance level was set at *P* ≤ 0.05, FWE svc.

## Results

### fMRI results

#### PIVC localizer results

**Figure 1:**
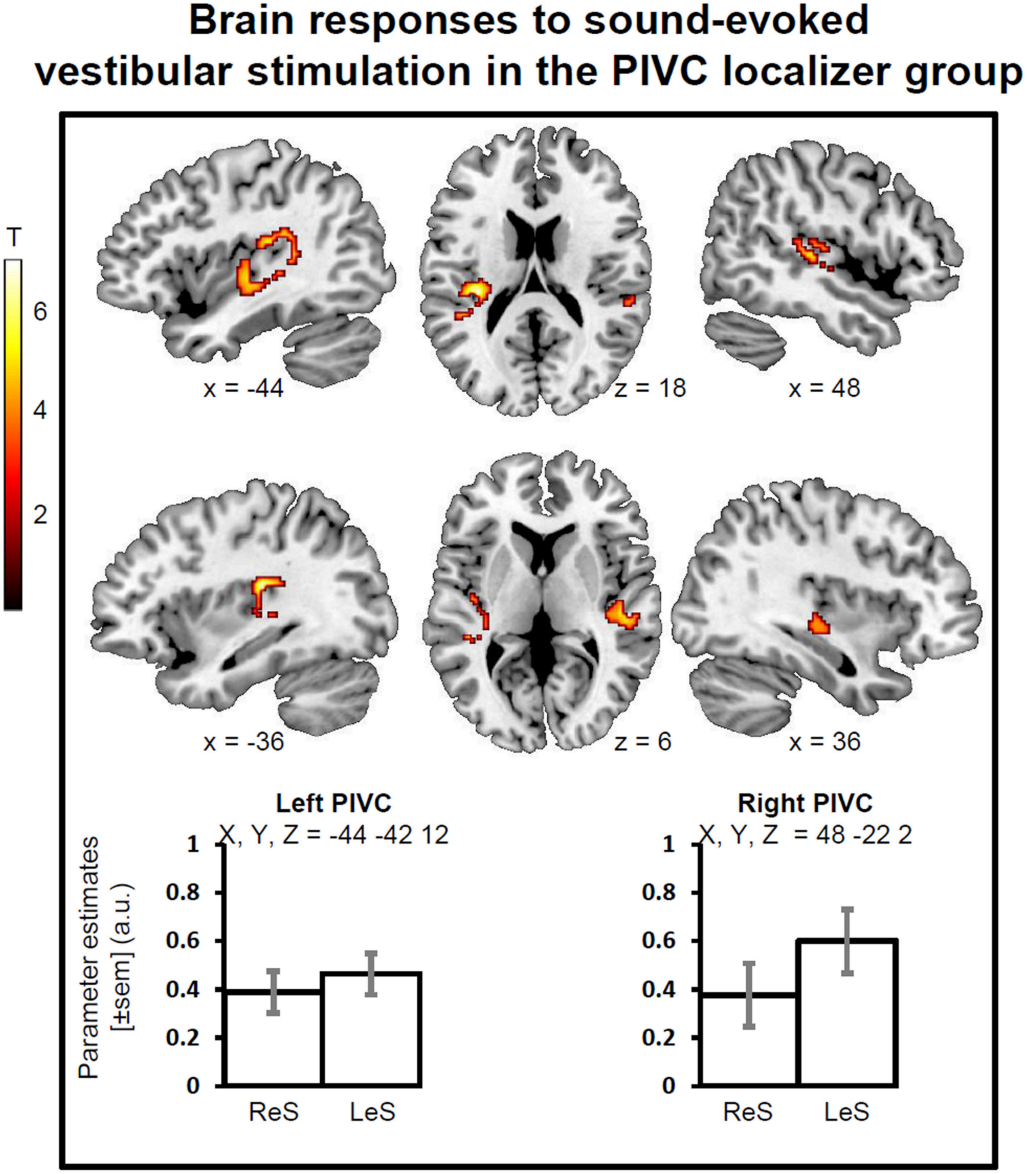
**Brain responses to sound evoked vestibular stimulation for the PIVC localizer group**. Statistical parametric maps (*p*-corr < 0.05, cluster level corrected for multiple comparisons) for the contrast of STB100 vs. STB65 exclusively masked for white noise. PIVC, Parieto-Insular vestibular cortex; ReS, Right ear Stimulation; LeS, Left ear Stimulation. Mean activity profiles (±sem) show the activation is bilateral for ReS and LeS.

During sound-evoked vestibular stimulation (STB100 dB > STB65 dB exclusively masked for white noise), the group of healthy participants showed robust activations in the left and right PIVC independently of the stimulation side, with the local maxima in the superior temporal gyrus (STg, MNI x, y, z = −44, −42, 12, Z-score = 4.71, p-FWE-CL corrected = 0.046, 197 voxels; MNI x, y, z = 48, −22, 2, Z-score = 4.41, p-FWE-CL corrected = 0.007, 305 voxels). The activations also encompassed part of the parietal operculum as well as the posterior insula (Figure 1).

#### Main effect of vestibular stimulation

**Figure 2:**
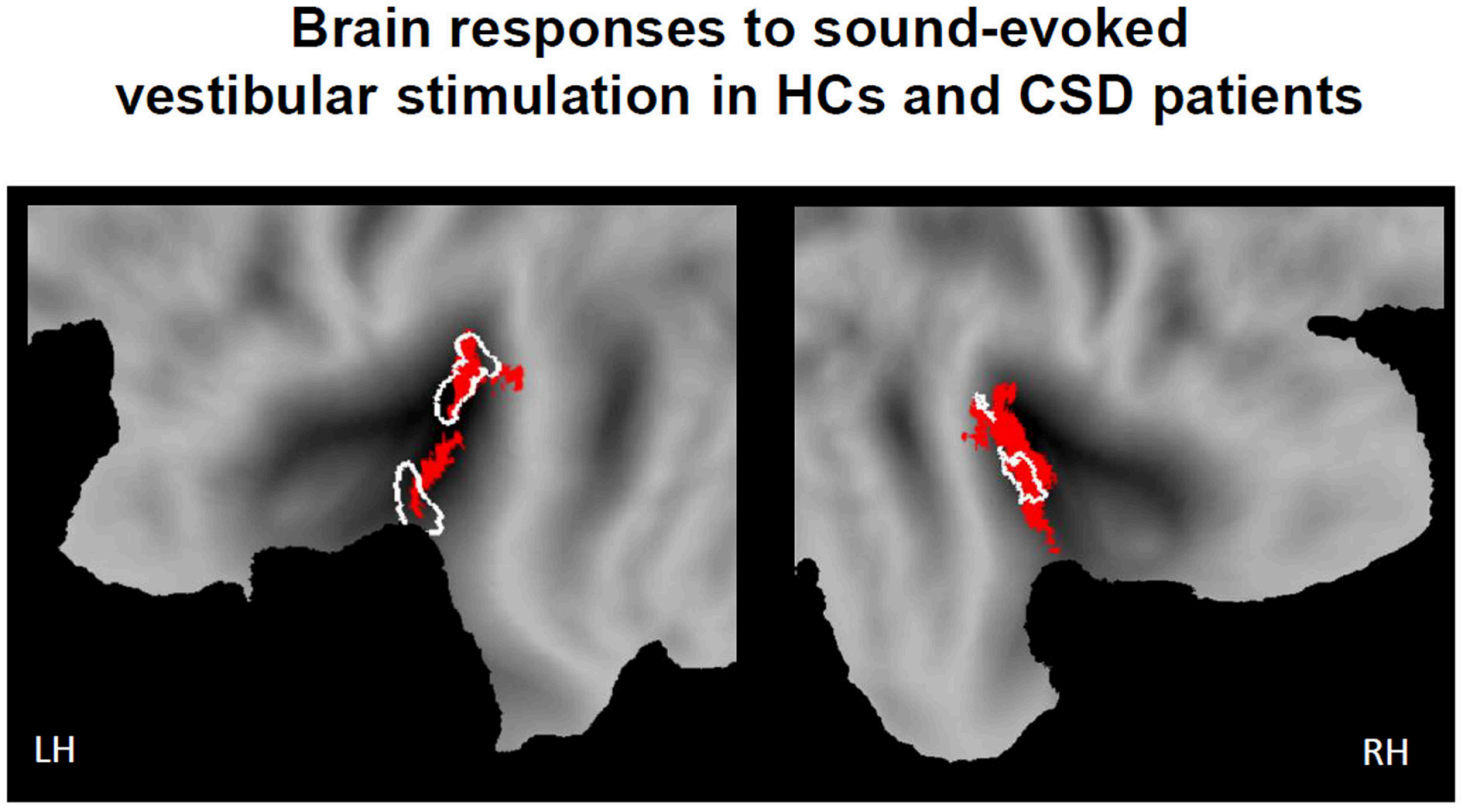
**Brain responses to sound evoked vestibular stimulation for the whole sample of participants (18 healthy controls and 18 CSD patients)**. Statistical parametric maps (*p*-corr < 0.05, cluster level corrected for multiple comparisons) for the contrast of STB100 vs. STB65 exclusively masked for white noise (above threshold *t*-values in red). Response of the PIVC localizer group is outlined in white for comparison. Response is projected onto flat maps of the left (LH) and right (RH) hemisphere of the human PALS atlas (Caret, Washington University School of Medicine, Department of Anatomy and Neurobiology, http://brainmap.wustl.edu).

During sound-evoked vestibular stimulation (STB100 dB > STB65 dB exclusively masked for white noise), the whole sample of participants (the 18 healthy controls and the 18 CSD patients) confirmed the results obtained in the localizer group. In particular, robust activations were found in the left and right PIVC independently of the stimulation side, with the local maxima in the superior temporal gyrus (STg, MNI x, y, z = −38, −20, 0, Z-score = 5.22, p-FWE-CL corrected = 0.004; MNI x, y, z = 40, −20, 2, Z-score = 4.82, p-FWE-CL corrected = 0.007). Again, the activations also encompassed part of the parietal operculum as well as the posterior insula (Figure 2).

#### CSD patients vs. healthy controls

Sound-evoked vestibular stimulation elicited significantly less activity in patients with CSD than in control subjects in a number of predefined ROIs. In particular, reduced activity in patients with CSD compared to healthy controls was found in the right posterior insula and adjacent superior temporal gyrus. Reduced activity in CSD patients relative to controls was also found in the left anterior insula extending to the adjacent frontal operculum in the left inferior frontal gyrus, bilateral hippocampus, and left cerebellar fastigial nuclei (Table 4, left columns).

**Table 4:** **List of brain regions showing reduced regional brain responses to short-tone burst vestibular stimulation in patients with chronic subjective dizziness (CSD) relative to healthy controls**.

	**Including people with psychiatric conditions**					**Excluding people with psychiatric conditions**				
	**P SVC**	***Z***	***x***	***y***	***z***	**P SVC**	***Z***	***x***	***y***	***z***
Posterior insula R	0.041	3.17	44	−2	−4	0.008	3.71	44	0	−6
PIVC (STg) R	0.025	3.11	44	−16	−4	0.01	3.43	44	−16	−4
Ant insula/IFg L	0.016	3.93	−44	12	6	0.046	3.73	−44	14	6
Hippocampus L	0.003	4.02	−28	−18	−20	0.009	3.74	−28	−18	−20
Hippocampus R	0.044	3.20	28	−16	−22	*(0.10)*	2.88	30	−16	−20
ACC L	*(0.08)*	3.08	−4	44	12	0.049	3.29	−6	44	12
Cerebellar fastigial nuclei L	0.02	3.03	−2	−52	−32	*(0.13)*	2.24	−2	−44	−26

*Areas met a threshold of P < 0.05, Family Wise Error, small volume correction for multiple comparison (except for the regions with a not significant P-value reported in Italics in brackets). Z, Z-score; L, left hemisphere; R, right hemisphere; x,y,z, MNI coordinates in millimeters; PIVC, Parieto-Insular Vestibular Cortex; STg, Superior Temporal gyrus; Ant Insula, Anterior Insula; IFg, Inferior Frontal gyrus; ACC, Anterior Cingulate Cortex*.

**Figure 3:**
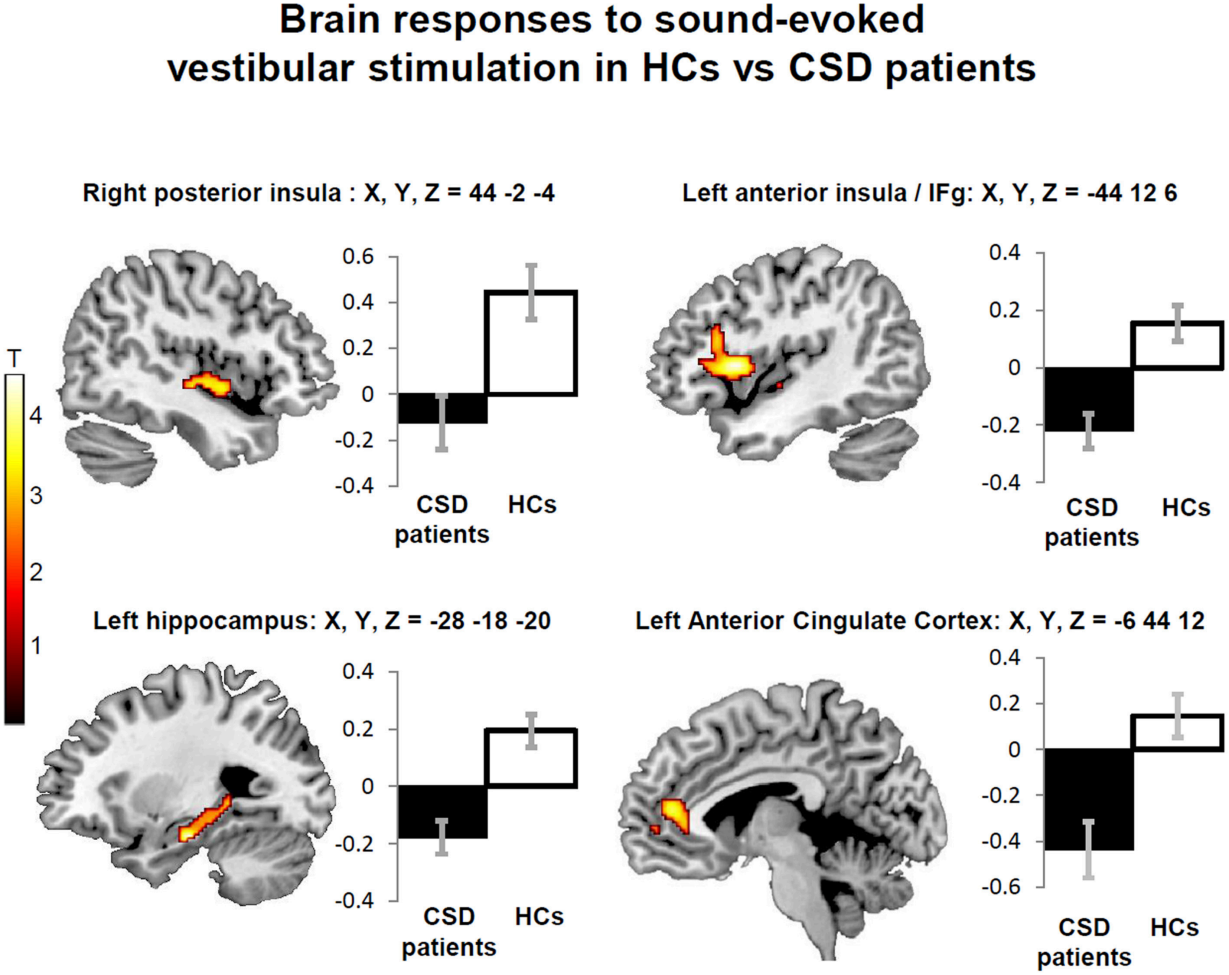
**Decreased regional brain activity in response to short-tone burst vestibular stimulation in patients with chronic subjective dizziness (CSD) relative to healthy controls (HCs)**. The regions displayed are those that were consistently found either when including or excluding participants with psychiatric comorbidities (posterior insula, anterior insula/inferior frontal gyrus, and hippocampus). Note also that an additional region (anterior cingulate cortex) was found only when excluding people with psychiatric comorbidities (Table 4). The coordinates (X,Y,Z) are in mm in the Montreal Neurological Institute space. Color bars represent T-statistics. Plots report parameter estimates ± SE in arbitrary units.

No significant main effect of stimulation side or any interaction between stimulation side and group was found, confirming the bilateral nature of the vestibular response to STB (Indovina et al., 2014). This also indicated that effects of CSD on brain activity alteration are independent of stimulation side. Follow-up analyses excluding people with active psychiatric conditions showed minimal effects of psychiatric comorbidity on the main results. Specifically, when 7 CSD patients and 1 control subject with active psychiatric disorders were removed from the analysis (see Methods; Figure 3; Table 4, right columns), only two areas (i.e., the right hippocampus and fastigial nuclei) were no longer significant (Figure 3; Table 4, right columns). On the other hand, the left anterior cingulate cortex showed reduced activation in CSD patients relative to healthy controls only when controlling for psychiatric comorbidity (Figure 3; Table 4, right columns).

#### PPI results

**Figure 4:**
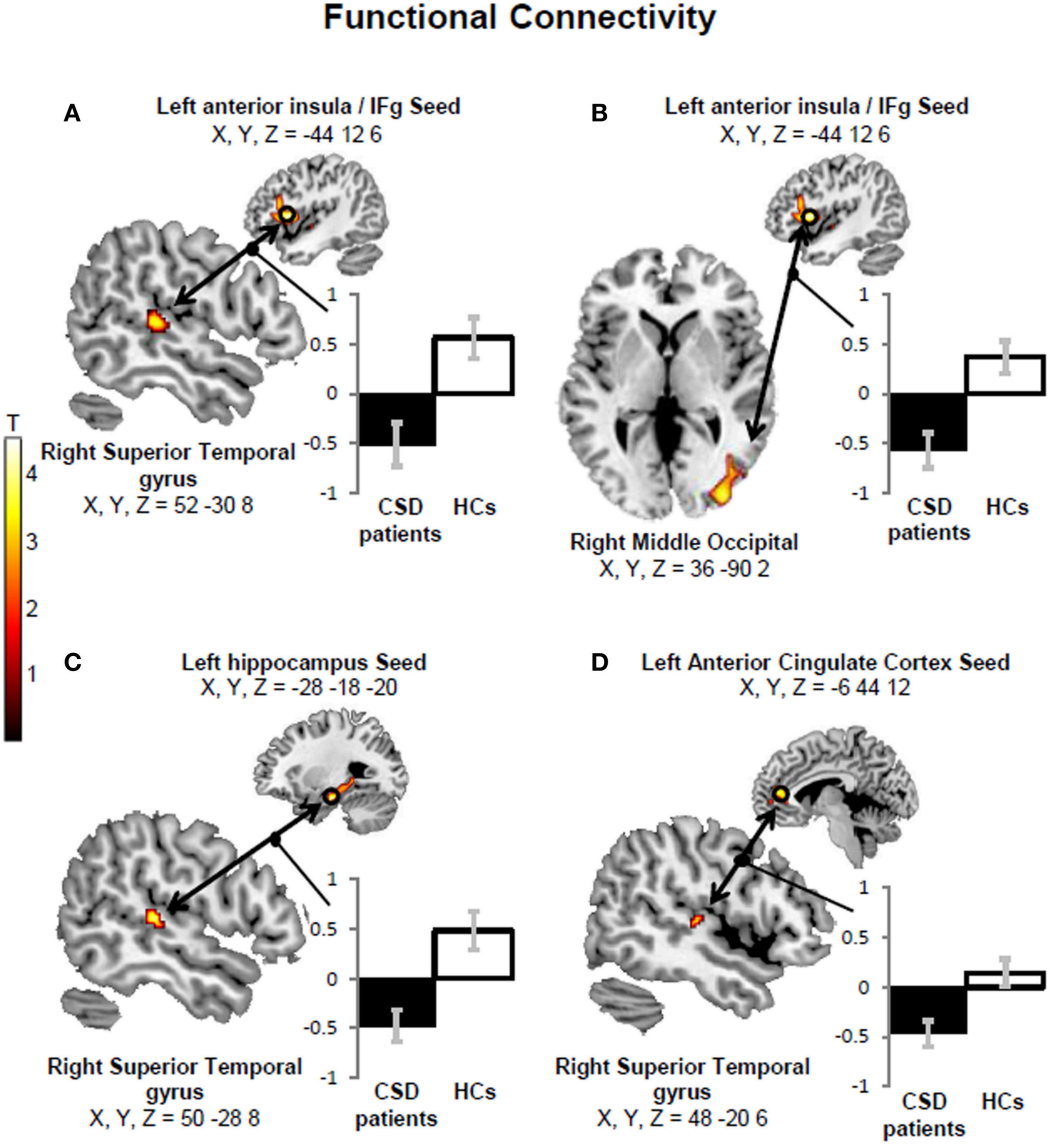
**A more negative functional connectivity change in response to short-tone burst vestibular stimulation was found in chronic subjective dizziness (CSD) patients relative to healthy controls (HCs) between the left anterior insula/inferior frontal gyrus (IFg) “seed” region and the right superior temporal gyrus (A)**. Likewise, more negative functional connectivity changes were found for the same comparison (i.e., HCs > CSD patients) between the left anterior insula/IFg and right middle occipital gyrus **(B)**; the left hippocampus and right superior temporal gyrus **(C)**; the left anterior cingulate cortex and right superior temporal gyrus **(D)**. The coordinates (X,Y,Z) are in mm in the Montreal Neurological Institute space. Color bars represent T-statistics. Plots report parameter estimates ± SE in arbitrary units.

**Figure 5:**
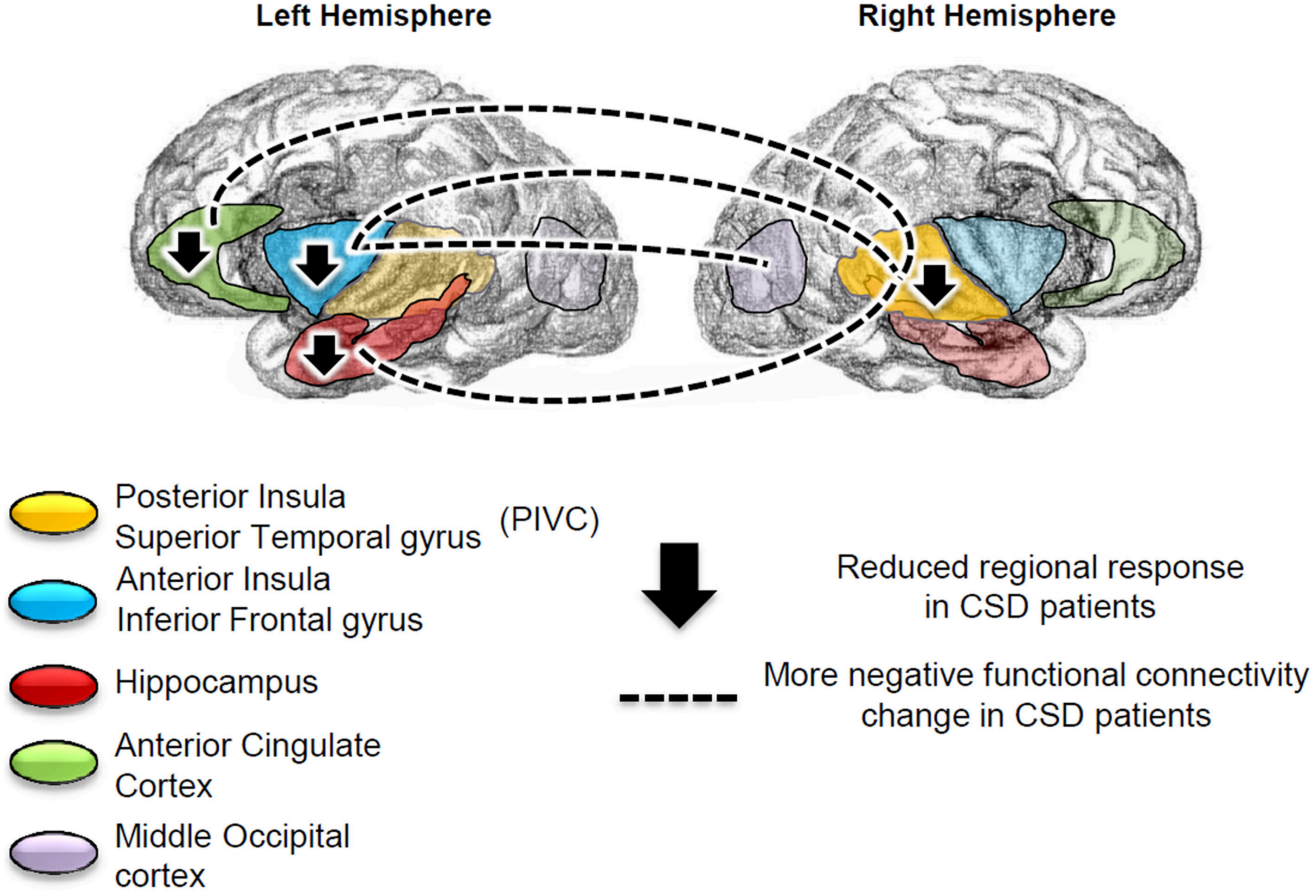
**Summary of the regional results and connectivity findings**. Patients with chronic subjective dizziness (CSD), relative to healthy controls, showed reduced brain responses to vestibular stimulation in the left anterior insula/inferior frontal gyrus, left hippocampus, left anterior cingulate cortex and right posterior insula. The left anterior insula more negative functional connectivity change with the right superior temporal gyrus [which is part of the Parieto-Insular Vestibular cortex (PIVC)], and with the middle occipital gyrus. Finally, the left anterior cingulate cortex and left hippocampus showed more negative functional connectivity changes with the right PIVC (superior temporal gyrus).

We found more negative functional connectivity changes between left anterior insula/inferior frontal gyrus (IFg) and right PIVC (superior temporal gyrus) (Figure 4A) and between the left IFg and right middle occipital cortex (Figure 4B) in CSD patients relative to controls. We also found more negative functional connectivity changes between the left hippocampus and right PIVC (superior temporal gyrus) (Figure 4C) (Table 5, left columns). The analysis excluding patients with active psychiatric conditions confirmed the results for the hippocampus-PIVC connectivity and anterior insula/IFg-occipital cortex connectivity. We also note that an additional connectivity effect (more negative connectivity change) was found between the left anterior cingulate cortex and right PIVC (superior temporal gyrus) (Figure 4D; Table 5, right columns) in CSD patients relative to controls, when participants with psychiatric comorbidity were excluded (see Figure 5 for a summary of results).

**Table 5:** **Brain regions showing a more negative functional connectivity change with the seed areas in patients with chronic subjective dizziness (CSD) relative to healthy controls**.

	**Including people with psychiatric conditions**					**Excluding people with psychiatric conditions**				
	**P SVC**	***Z***	***x***	***y***	***z***	**P SVC**	***Z***	***x***	***y***	***z***
Seed: ant insula/ IFg L target: PIVC (STg) R	0.017	3.27	52	−30	8	*(0.055)*	2.85	52	−28	6
Seed: ant insula/ IFg L target: middle occipital R	0.033	3.53	36	−90	2	0.005	4.14	36	−90	2
Seed: hippocampus L target: PIVC (STg) R	0.003	3.83	50	−28	8	0.049	2.92	54	−26	6
Seed: ACC L target: PIVC (STg) R	*(0.14)*	2.57	46	−20	6	0.046	2.92	48	−20	6

*Areas met a threshold of P < 0.05, Family Wise Error, small volume correction for multiple comparison (except for the regions with a not significant P-value reported in Italics in brackets). Z, Z-score; L, left hemisphere; R, right hemisphere; x,y,z, MNI coordinates in millimeters; IFg, Inferior Frontal gyrus; Ant Insula, Anterior Insula; PIVC, Parieto-Insular Vestibular Cortex; STg, Superior Temporal gyrus; IFg, Inferior Frontal gyrus; ACC, Anterior Cingulate Cortex*.

## Discussion

We found that patients with CSD showed decreased regional activity and more negative patterns of functional connectivity relative to healthy controls in response to sound-evoked vestibular stimulation in key regions of the vestibular, visual and anxiety systems. In particular, patients with CSD showed reduced activation of posterior and anterior insula and adjacent frontal operculum in the inferior frontal gyrus, hippocampus, and anterior cingulate cortex. They also showed more negative functional connectivity changes between anterior insula/inferior frontal gyrus and PIVC, anterior insula and middle occipital cortex, hippocampus and PIVC, and anterior cingulate cortex and PIVC.

PIVC is the core vestibular cortical region and is involved in processing vestibular inputs, self-motion perception, estimation of verticality as well as processing visual motion, particularly motion coherent with gravity (Penfield, 1957; Cardin and Smith, 2010; Maffei et al., 2010, 2015; Indovina et al., 2013; Lacquaniti et al., 2013; zu Eulenburg et al., 2013; Mazzola et al., 2014; Shinder and Newlands, 2014). As cardinal symptoms of CSD are aberrant sensations of self-motion, a relative inactivation of PIVC in CSD patients makes it a potential site for the generation of these symptoms. Perhaps more importantly, altered signaling between the PIVC and anterior insula, which is thought to integrate bottom-up interoceptive signals with top-down predictions to generate a body-centered awareness state (Craig, 2009; Gu et al., 2013), may impair production of a stable sense of self-motion.

Negative connectivity changes between prefrontal regulatory areas and PIVC as well as visual areas may reflect alterations in normal inhibitory interactions between vestibular and visual areas in patients with CSD. These interactions are thought to be cortical adaptive mechanisms which are responsible for stabilization of visual perception during self-motion. Over-reliance on visual information for spatial orientation (i.e., visual dependence) has been correlated with persistent CSD-like dizziness following bouts of acute vestibular neuritis (Cousins et al., 2014). The negative connectivity change between visual cortical areas and anterior insula may therefore subtend altered top-down signal to visual areas from the anterior insula, and could represent the neural correlate of visual dependence in CSD.

The results of this study also offer insights into the role of anxiety-related mechanisms in sustaining CSD. In fMRI studies of patients with chronic post-traumatic stress disorder, the trauma-related re-experiencing of symptoms were linked to hypoactivation of the hippocampus, anterior cingulate cortex, and anterior insula, as well as to weaker connectivity across these structures (Etkin and Wager, 2007; Suvak and Barrett, 2011; Spielberg et al., 2015). The hippocampus also plays a key role in contextual tuning of memories (LeDoux, 2000; Grillon, 2002; Alvarez et al., 2008), and together with the anterior cingulate cortex, it promotes effective extinction of conditioned responses to noxious stimuli (Kalisch et al., 2006; Ji and Maren, 2007; Indovina et al., 2011; Baldi and Bucherelli, 2015). Furthermore, hippocampal hypofunction in patients with CSD may reduce their ability to place space-motion stimuli in proper context at the same time that reduced activity of the anterior insula and anterior cingulate cortex impair their ability to assess the relevance of this information. These anterior structures along with the frontal operculum have a role in assessing salience of sensory stimuli (Seeley et al., 2007). This could explain why patients with CSD perceive routine space-motion stimuli encountered during normal daily activities as highly challenging.

Though CSD patients generally show higher levels of neuroticism and introversion and greater psychiatric comorbidity than healthy people (Staab et al., 2014), our results appeared independent of these factors. In addition, in a previous study in healthy volunteers, individual differences in neuroticism and introversion were found to be correlated with different brain regions than those identified here (Indovina et al., 2014). However, the brain regions showing altered patterns of activity and connectivity in the present study of patients with CSD did not overlap with areas that correlated with personality traits in healthy individuals in the previous investigation. Hence, the results of this study may reflect the neural basis of the core elements of CSD.

In summary, disruption of key vestibular-visual-anxiety network was found in patients with CSD. This network integrates information from vestibular inputs (mainly processed in the PIVC at the cortical level) with information about the space-motion context in which individuals live and work (which is primarily dependent on hippocampal function). These functional changes may be associated with the primary CSD symptoms of persistent unsteadiness and dizziness as well as sensitivity to postural changes and body motion. Altered connectivity between frontal regions and visual cortices may further impair sensory integration, leaving patients vulnerable to visually induced dizziness. Finally, hypofunction, and altered connectivity between anterior cortical structures that play a role in anxiety modulation (anterior insula, ACC) and the PIVC may maintain the disorder by impeding the return to normal, low-threat postural strategies. Understanding the circuits involved in CSD represents an important challenge for improving definitions of the disorder and developing new therapeutic approaches.

### Conflict of interest statement

The authors declare that the research was conducted in the absence of any commercial or financial relationships that could be construed as a potential conflict of interest.
